# Dendritic cells in hematologic malignancies: a brief review of immunoregulation to new perspectives

**DOI:** 10.11613/BM.2026.020501

**Published:** 2026-04-15

**Authors:** Gabriel Barroso de Almeida, Mariane Melo dos Santos, Dyjaene de Oliveira Barbosa, Rogério Reis Conceição, Bruno de Almeida Lopes, Herbert Henrique de Melo Santos, Silvana Beutinger Marchioro

**Affiliations:** 1 Laboratory of Immunology and Molecular Biology, Institute of Health Sciences, Federal University of Bahia, Bahia, Brazil; 2 Laboratory of Acute Leukemia Genetics, National Cancer Institute, Rio de Janeiro, Brazil

**Keywords:** dendritic cells, plasmacytoid dendritic cell, acute myeloid leukemia

## Abstract

Dendritic cells (DCs) are essential immune system sentinels, playing a key role in activating naïve T cells against leukemic cells. They have an immunomodulatory function, promoting an immune response and, at the same time, regulating immune tolerance to avoid excessive reactions. Plasmacytoid dendritic cell (pDC) neoplasms are a complex group of disorders, and among the subtypes, acute myeloid leukemia (AML) with pDC dermatosis (AML-pDC) stands out. In this context, pDCs can play an immunomodulatory role by inhibiting or promoting immune responses and acting as tumor progenitors since, in some cases, they can collaborate with the tumor microenvironment, favoring immune evasion and proliferation. However, the clinical significance of pDC in AML is not fully understood. Studies suggest that the interaction of pDCs with the leukemic microenvironment may contribute to disease progression by impairing the immune system’s ability to control tumor cells. Understanding the role of DCs in neoplasia is crucial, as it provides important insights for new studies seeking advances in innovative therapeutic strategies, such as dendritic cell vaccines. These vaccines are promising, with low toxicity and the potential to improve the prognosis of individuals affected by AML, stimulating an effective immune response.

## Graphical abstract

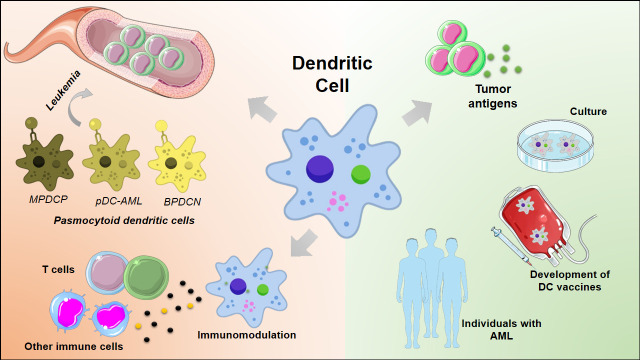

## Highlights

• Classification of plasmacytoid dendritic cell-related hematologic neoplasms

• Immunomodulatory role of dendritic cells in the leukemic microenvironment

• Therapeutic advances in acute myeloid leukemia through dendritic cell-based vaccination strategies

## Introduction

Immunological aspects play a crucial role in the development and progression of malignant hematological diseases, and recent research has shed increasing light on the role of innate immune cells, particularly within leukemic contexts (*1*, *2*). Among these, dendritic cells (DCs) have received considerable attention. While they play a pivotal role in initiating and regulating immune responses against tumor cells, accumulating evidence also highlights their potential to support tumor progression, particularly through the induction of immunosuppressive microenvironments (*3*). This duality reinforces the complexity of DCs in leukemogenesis and underscores the need for a deeper understanding of their functional plasticity.

In parallel with functional studies, advances in immunophenotyping, molecular profiling, and genetic analyses have redefined DC-related disorders in hematologic malignancies (*4*, *5*). The expansion of neoplastic dendritic cells has long been recognized in these diseases; however, recent classifications reflect a more refined understanding of their biology (*6*). Among the dendritic cell subsets, plasmacytoid dendritic cells (pDCs) have emerged as particularly relevant in myeloid neoplasms (*7*). Currently, three distinct types of neoplastic pDC proliferation are described: 1) blastic plasmacytoid dendritic cell neoplasm (BPDCN), 2) acute myeloid leukemia with pDC differentiation (pDC-AML), and 3) mature plasmacytoid dendritic cell proliferation (MPDCP). Each entity exhibits distinct phenotypic features that correlate with different stages of pDC maturation, and these features have significant clinical and therapeutic implications (*8*-*10*).

Given this constantly evolving scenario, this descriptive review searched databases such as PubMed and SciELO, including original articles, literature reviews and experimental studies, using descriptors such as pDC, AML, immunotherapy, and vaccine. The objective was to provide an updated overview of dendritic cells in leukemic contexts, with an emphasis on their classification, immunological functions, and functional heterogeneity. Furthermore, it sought to investigate how a deeper understanding of the biology of these cells, particularly the immunomodulatory properties of pDCs, can inform the development of targeted immunotherapeutic strategies, leading to a more precise characterization of the disease and the advancement of innovative therapeutic approaches.

## Dendritic cells subsets and biology

**Figure 1:**
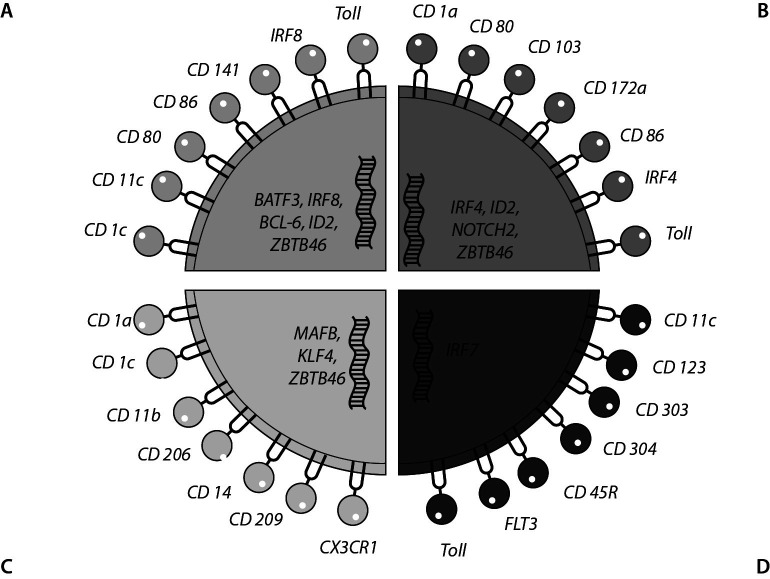
Main dendritic cell (DC) subsets, their corresponding cellular markers, and the associated transcription factors that drive their development. **A.** Conventional DC type 1 IRF (interferon regulatory factor), and TLR (Toll-like receptors), transcription factors such as BATF3 (Basic Leucine Zipper ATF-Like Transcription Factor 3), IRF8, BCL-6 (B-cell lymphoma 6); ID2 (Inhibitor DNA Binding 2); ZBTB-42 (Zinc Finger and BTB Domain Containing 42). **B.** Conventional DC 2 has four transcription factors: IRF4, ID2, ZBTB-46, and NOTCH2 (notch receptor 2). **C.** Monocyte-derived DC surface CX3CR1 (CX3C chemokine receptor), transcription factors MAFB (MAF transcription factor B), KLF4 (KLF transcription factor 4). **D.** Plasmacytoid DC IRF7. Figure created using Servier Medical Art resources (Servier Medical Art, CC BY 4.0 license).

Dendritic cells comprise a heterogeneous group characterized by diverse locations, phenotypes, and immunological functions, with a standard function in antigen presentation. These cells possess numerous pattern recognition receptors (PRRs). Known as professional antigen-presenting cells (APCs), they are most effective at activating naïve T cells through MHC-I for CD8+ T cells and MHC-II for CD4+ T cells (*11*, *12*). The main subsets described are shown in Figure 1. This plasticity enables DCs to shape the immune response differently when encountering various pathogens (*4*).

Under homeostatic conditions, DCs are present in various tissues and can migrate to the T cell zones of lymph nodes during the inflammatory process. Depending on the stimulus, DCs alter their phenotypes, which can exert either immunogenic or tolerogenic effects (*4*). Conventional type 1 DCs (DC1) are located in the paracortex of lymph nodes and capture cellular antigens through CD205+ or TIM3 (mucin domain-containing protein 3) receptors (*12*). They facilitate the presentation of cross-antigens to CD4+ and CD8+ T cells. DC1 are equipped with toll-like receptors (TLR) 3, 4, 9, 11, and 13, and primarily produce interleukin (IL) 12 and interferon (IFN) III, which are critical in the responses against viruses, tumors, and intracellular bacteria (making them the most efficient antigen-presenting cells for stimulating the Th1 phenotype) as well as for regulatory T cells (*12*, *13*). Data demonstrates that in Batf3^+^/^+^ mice, deficient in DC1, there is a failure in the migration of CD8+ T lymphocytes. Replacement with activated DCs from Batf3^+^/^+^ mice restores this response, indicating that only CD103+ DC1s are indispensable and cannot be replaced by other DC subtypes. However, their antitumor role may be limited, for example, by the action of granulocyte colony-stimulating factor (G-CSF), which suppresses the expression of interferon regulatory factor 8 (*IRF8*), an essential factor for the development of DC1 (*14*).

Conventional type 2 DCs (DC2) participate in the capture of antigens in the skin and migrates to the T zones of lymph nodes through chemoreceptors (CXCR4 and CCR7). They are cells that participate in presenting tumor-associated antigens (TAAs). In addition, DC2 expresses interferon regulatory factor 4 (IRF4), which enables them to present MHC-II efficiently and is more effective in activating CD4+ T cells compared to DC1 (*4*, *15*). They are endowed with TLR (*1*, *4*-*7*, *9*, *13*) and mainly express tumor necrosis factor (TNF), IL-6, and IL-23, which are associated with responses to extracellular bacteria, fungi, and allergens, and are specialized in activating T for Th2 and Th17 subtypes (*15*).

Conventional type 2 DCs are considered the most heterogeneous subset of DCs both phenotypically and functionally (*16*). Single-cell transcriptome analyses have revealed that human DC2s are subdivided into at least two main groups: cDC2 and DC3. In mice, inflammatory cDC2s (inf-cDC2s) have been identified, characterized by the expression of CD172a and CD11b. Notably, these inf-cDC2s also express CD64 and IRF8, typical markers of monocyte-derived DC (moDCs) and cDC1s, respectively. Studies demonstrate that TLRs and type I IFN promote the maturation of these inf-cDC2s in an IRF8-dependent manner (*17*, *18*).

Recently described DC subgroups, such as immature DCs (imDCs) and regulatory DCs (DCregs), have been implicated in the formation of an immunosuppressive microenvironment that favors tumor progression. A new profile of CD11c+ MHCII^low^ DCs (T-DCs) has recently been identified, accumulating in the splenic microenvironment of mice with T-cell acute lymphoblastic leukemia (T-ALL). These T-DCs exhibited an immature phenotype, characterized by low expression of MHC II, CD86, CD83, and CD40 molecules. Functionally, T-DCs promoted T-ALL progression, exhibiting reduced phagocytic capacity and limited potential for activating CD4+ T cells. Furthermore, RNA analyses demonstrated that T-DCs express low levels of genes associated with maturation and antigen processing. Single-cell RNA sequencing studies further revealed the heterogeneity of these cells, showing that they are predominantly composed of DC1s, DC2s, and DCs with macrophage-like characteristics (*19*). Thus, the heterogeneity observed among the DC subsets appears to reflect transcriptional profiles shaped by different immunological and environmental stimuli, highlighting the importance of understanding the differentiation factors and pathways that drive the development of these populations.

Inflammatory or moDCs arise in inflammatory contexts and are present in the blood, mucous membranes, lymphoid organs, and inflamed tissues. They express multiple TLRs (*1*, *2*, *4*, *6*-*9*, *13*), produce pro-inflammatory cytokines (TNF, iNOS, IL-12, and IL-23), and promote Th1 responses through cross-presentation of antigens to CD8+ and CD4+ T lymphocytes (*4*, *15*). In mice, moDCs can present antigens directly in peripheral tissues, activating both naive and memory CD8+ T lymphocytes, as observed in models of acute lymphocytic choriomeningitis virus infection, where a drastic increase in moDCs is seen in secondary lymphoid organs in an IFN-γ-dependent manner. In these models, activation by moDCs enhances the differentiation of memory CD8+ T cells compared to cDC, through the upregulation of Eomesodermin (Eomes) and TCF-1 (*20*). Although this function is not yet fully understood in humans, evidence suggests similar mechanisms, including cross-presentation *via* the vacuolar pathway and activation of CD8+ T lymphocytes in non-lymphoid tissues by moDCs derived from ascites fluid (*21*).

Among the methodologies employed in therapeutic strategies involving DCs, the use of moDCs stands out. In various types of cancer, such as melanoma, and in leukemia, where leukemic DCs may be dysfunctional, autologous or allogeneic moDCs become a preferred alternative (*22*). One limitation is that moDCs do not naturally express leukemic antigens, making it necessary to load them *ex vivo* (*23*). However, data demonstrate that these moDC vaccines can induce effective antigen-specific responses against leukemic clones (*24*).

Plasmacytoid DCs (pDCs) are found in the bone marrow, secondary lymphoid organs, and blood but have limited antigen presentation capacity. Throughout their development, they present transcription factors such as *E2-2, ZEB2, IKZF1, MTG16*, and during their maturation, genes such as *RUNX2, SPIB, IRF7, and NFATC3* (*15*). They play a crucial role in responses to RNA and DNA viruses, as they express TLRs 7, 9, and 12. When activated, they release type I and III interferons, which have activity under NK and activate the antiviral state in host cells. In addition to their well-known antiviral activities, pDCs have been shown to play a fundamental role in the development of various inflammatory diseases (*4*, *11*).

## Role of pDC in acute myeloid leukemia

Studies on the role of plasmacytoid dendritic cells (pDCs) in leukemias have gained increasing attention in recent years. Although this cell group comprises less than 1% of nucleated cells in the bone marrow and blood, it is recognized as the primary producer of type I and III interferons (*25*). Conventionally, pDCs are identified by the expression of CD123, HLA-DR, and CD303/BDCA2, in the absence of other lineage-specific markers (*e.g.*, lymphoid and/or myeloid), and also express the FLT3 cytokine receptor (*12*, *26*).

Given their potent capacity to produce interferons, particularly types I and III, pDCs have been considered key players in anti-tumor immunity. Interferons can promote the death of cancer cells *via* cytotoxic mechanisms; however, depending on the stimuli, pDCs may adopt a pro-tumorigenic phenotype. For instance, impaired IFN production can foster a microenvironment conducive to Treg expansion, which may support tumor growth (*3*, *27*).

This duality in pDC function is further illustrated by their role in modulating T cell responses. In solid tumors, a higher density of dendritic cells within the tumor microenvironment is associated with enhanced activation of CD8+ T cells and, consequently, a reduction in neoplastic clones (*28*). Interestingly, during acute myeloid leukemia (AML), pDCs typically remain in a quiescent state. However, stimulation with TLR7 and TLR9 agonists has shown promise in reprogramming their tolerogenic profile. This reprogramming enhances endogenous type I IFN production and may facilitate the clearance of leukemic cells (*29*). Supporting this, murine models have demonstrated that antigens from circulating leukemic cells can be captured by a subset of splenic DCs, known as CD8α+ DCs, which facilitate the presentation of tumor antigens to CD8+ T cells, thereby promoting an anti-leukemic response (*30*).

In the context of myelodysplastic syndromes (MDS), the role of pDCs is also gaining prominence. Studies have shown that lower proportions of pDCs correlate with poorer prognoses, as classified by the International Prognostic Scoring System-Revised (IPSS-R), and are associated with decreased patient survival (*31*). These findings highlight the potential of pDCs to serve as biomarkers that complement traditional clinical risk stratification. Furthermore, reduced pDC counts in grafts have been linked to higher incidences of acute graft-*versus*-host disease (aGVHD) in pediatric patients undergoing allogeneic hematopoietic stem cell transplantation. In this context, low peripheral blood pDC counts may also predict transplant-related mortality, reinforcing their clinical relevance (*32*).

## Neoplasms involving pDCs

The importance of pDCs is further emphasized by their involvement in plasmacytoid dendritic cell neoplasms, highlighting their ambiguous role in the tumor microenvironment. It is hypothesized that pDCs originate from common hematopoietic progenitors (CD34+), derived from monocytes (CD14+) and conventional dendritic cells (*15*, *33*). However, they may also derive from leukemic clones in AML and MDS, suggesting a complex interaction between leukemic cells and the pDC progeny (*26*, *27*). In this context, the observation that pDCs are markedly depleted in most cases of AML and other high-grade myeloid neoplasms, while their increased differentiation may point to an early hematopoietic stem cell or multipotent progenitor as the cell of origin, reinforces the notion that pDC dysregulation is intricately linked to disease pathogenesis and may serve as an indicator of adverse clinical outcomes (*26*).

**Figure 2:**
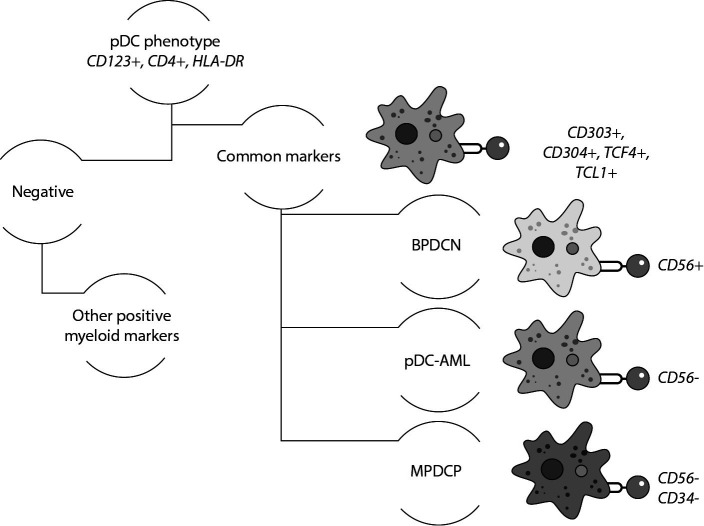
Main immunophenotypic markers used in the diagnosis of malignant dendritic cell neoplasms. Positive phenotype for pDC lineage (CD123+, CD4+, HLA-DR) and common markers (CD303+, CD304+ TCF4+ TCL1+). BPDCN (Blastic plasmacytoid dendritic cell neoplasm) is identified by the expression of CD56+; MPDCP (Mature plasmacytoid dendritic cell proliferation) presents CD56- and CD34-; pDC-AML (Acute Myeloid Leukemia with Plasmacytoid Dendritic Cell Differentiation) presents CD56-. Figure created using Servier Medical Art resources (Servier Medical Art, CC BY 4.0 license).

Throughout their development, pDCs progress through three maturation stages: early, intermediate, and late, each characterized by distinct marker expression profiles. Early-stage pDCs express CD34 and CD117 while lacking CD303 and CD304. As they transition to the intermediate stage, they begin to express CD4, CD303, and CD304, while concurrently declining in CD34 and CD117 expression. In the final stage, mature pDCs express CD4, CD303, CD304, and CD45 (Figure 2). Notably, CD123 and HLA-DR are consistently expressed across all stages and serve as reliable markers for both mature and immature pDCs. Additionally, CD56 expression may appear during intermediate stages, particularly when CD2 and CD5 are co-expressed (*34*, *35*).

Myeloid neoplasms with pDC differentiation, also described as pDC expansion in myeloid neoplasms (MN), are commonly associated with acute myeloid leukemia (pDC-AML). Representing approximately 3-5% of all AML cases, these neoplasms exhibit a median pDC proportion of 6.6%. Unlike other AML subtypes, pDC-AML can present with varying degrees of pDC maturation, often in early or intermediate stages (*26*). This feature reflects the abnormal differentiation trajectory of pDCs previously discussed. Notably, pDC-AML is also observed in AML subtypes with higher cellular differentiation, such as M4 (acute myelomonocytic leukemia) and M5 (acute monocytic leukemia), and typically affects individuals between 45 and 70 years of age (*10*). Clinical manifestations are diverse and may include anemia, thrombocytopenia, lymphadenopathy, and cutaneous lesions. Skin involvement appears more frequent in pDC-AML than in blastic plasmacytoid dendritic cell neoplasm (BPDCN). Importantly, patients with pDC-AML tend to have lower complete remission rates and a reduced median overall survival of approximately 8 months, compared to other AML cases (*10*, *35*).

Building upon the clinical profile, genetic and epigenetic studies have begun to uncover molecular characteristics of pDC-AML, although research in this area remains limited. An influential association has been identified with *RUNX1* mutations, which affect the RUNT DNA-binding domain and are found in approximately 78% of pDC-AML cases. These mutations are less frequent in MPDCP and appear to drive leukemogenesis by promoting the expansion and differentiation of pDCs from leukemic blasts (*36*, *37*). This mechanism reinforces the idea that pDCs in pDC-AML may not be reactive or bystanders but derive directly from the leukemic clone. Other recurrent mutations associated with pDC-AML include *TP53, TET2, ASXL1,* and *DNMT3A*; however, mutations in *FLT3 I/D*, although commonly observed, are not considered unique to this AML subtype (*38*).

Compared to BPDCN, it presents a distinct but overlapping molecular landscape. Recent genome-wide analyses have shown significant deregulation of key regulatory pathways in BPDCN, including a marked loss of DNA methylation, indicative of mitotic stress and epigenetic instability (*39*). This contrasts with the genetic profiles seen in pDC-AML, highlighting divergent pathophysiological mechanisms even among neoplasms involving pDCs. In BPDCN, differentially expressed genes include *STAT5A, CDK6, CCR4, CCND2,* and *FOXO1*, which contribute to a molecular profile distinct from other dendritic cell disorders. Structural chromosomal abnormalities, such as deletions or rearrangements in regions like 5q, 12p, 17p, and monosomy 9, further characterize BPDCN. Moreover, gene-level mutations commonly involve *ASXL1, IDH1/2, TET1/2, TP53*, and others (*35*, *38*). The elevated expression of genes such as *LAMP5* and *CCDC50*, alongside increased levels of eosinophilic chemoattractant cytokines (*e.g*., eotaxin, RANTES), suggests a possible pre-inflammatory microenvironment, despite pDCs in BPDCN often displaying a non-activated phenotype (*35*).

## Immunosuppressive mechanisms

These molecular and immunological changes underscore a broader phenomenon observed across dendritic cell-related neoplasms - immune dysregulation within the tumor microenvironment (TME). In AML, for example, the TME has been shown to promote the conversion of innate immune cells to an immunosuppressive phenotype, contributing to tumor progression (*3*, *40*). RNA-seq analyses of bone marrow samples from AML patients have revealed a highly heterogeneous and altered immune landscape, including the presence of Th17 cells, CD8+ memory T cells, exhausted T lymphocytes, regulatory T cells (Tregs), macrophages, and dysfunctional dendritic cells (*41*). These findings reinforce the notion that pDCs and other DC subsets, whether as part of the leukemic clone or as modulators of the immune response, play a central role in reshaping the AML microenvironment and may serve as biomarkers and potential therapeutic targets.

The obstacles to an effective immune response in AML are multifactorial and contribute significantly to the disease’s capacity to evade immune surveillance. These obstacles include: i) deficient antigen presentation due to low neoantigen load; ii) imbalance in T cell subpopulations, particularly an increase in Tregs; iii) T cell exhaustion caused by chronic inflammatory stimulation and overexpression of inhibitory ligands; iv) expansion of myeloid-derived suppressor cells (MDSCs) and tumor-associated macrophages (TAMs); and v) increased production of immunomodulatory factors and metabolic mediators (*42*).

Within this context, AML is broadly considered a malignancy of low immunogenicity (*42*). Neoplastic clones in AML downregulate genes and proteins related to MHC class II (such as HLA-DP and HLA-DR), impairing antigen presentation by dendritic cells (DCs) to naïve T cells (*43*). DC-AML fusion vaccines have emerged as a promising therapeutic approach to counteract this immunoevasive strategy. These fusion cells can present a broader array of epitopes and activate multiple T cell receptors (TCRs), potentially restoring effective immune responses.

The immunosuppressive environment in AML is further compounded by the prominent role of Tregs, which can inhibit various immune system components. Elevated levels of Tregs are commonly reported, particularly after chemotherapy, and their abundance is associated with delayed hematopoietic recovery. Consequently, strategies targeting Tregs have gained interest. Additionally, T cell exhaustion, characterized by the expression of co-inhibitory receptors such as CTLA-4, PD-1, TIM-3, and LAG-3, has been described in AML. Leukemic blasts can express PD-L1/PD-L2 in response to interferon signaling, further dampening effector T cell responses (*3*, *40*).

This immune dysfunction is compounded by MDSCs and TAMs, both of which adopt pro-tumor phenotypes under the influence of the leukemic microenvironment. These cells, together with immunosuppressive mediators such as arginase pathway products, indoleamine 2,3-dioxygenase (IDO), and prostaglandin E2 (PGE2), contribute to the increased recruitment of Treg cells and further suppression of anti-tumor immunity (*2*, *44*, *45*). These molecules also promote programmed death-1 (*PD-1*) expression, reinforcing the exhausted phenotype of T cells.

At the metabolic level, AML blasts have been shown to overexpress *STAT5*, a transcription factor that enhances glycolytic metabolism. The resulting lactate accumulation in the bone marrow can promote nuclear translocation of E3-binding protein (E3BP), facilitating histone acetylation and upregulating programmed death-ligand 1 (*PD-L1*) transcription. This metabolic reprogramming has been clinically correlated with increased STAT5 and PD-L1 levels in newly diagnosed AML patients, further linking metabolic alterations with immune evasion (*46*).

**Figure 3:**
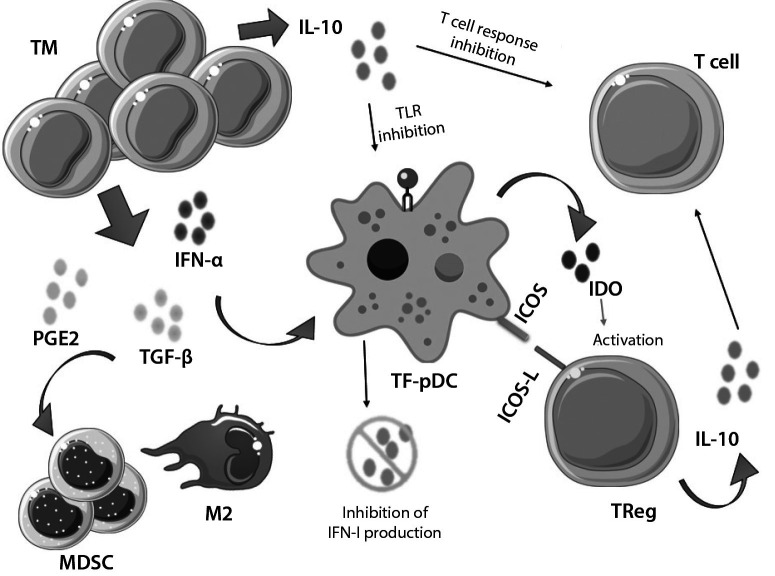
Main mechanisms involved in DC-mediated immunosuppression in hematologic malignancies. Produced by TM, cytokines such as PGE2, TGF-β, and TNF-α can stimulate the development of MDSC and M2 macrophages, thereby inhibiting inflammation. These cytokines lead to low IFN production in TF-pDC. TF-pDCs are associated with the development of Tregs. IL-10 produced by TM or TReg causes inhibition of the T response and low expression of Toll-like receptors in DC. The production of IDO and expression of ICOS and its ligands stimulate the differentiation of T cells in an anti-inflammatory profile.TM - tumor microenvironment. IL-10 - interleukin 10. IFN-α - interferon alpha. TGF-β - transforming growth factor beta. PGE2 - prostaglandin E2. MDSC - Myeloid-Derived Suppressor Cells. M2 - Macrophage M2. TLR - Toll-like receptors. TF-PDC - tumor-forming pDCs. ICOs - Inducible costimulator-ligands. Figure created using Servier Medical Art resources (Servier Medical Art, CC BY 4.0 license).

Amid these alterations, pDCs have been implicated in immune modulation and tumor progression. Recent studies describe a unique subset termed tumor-associated pDCs (TAPDCs) or tumor-forming pDCs (TF-pDCs). Some mechanisms related to this process are illustrated in Figure 3; among them, these cells, often expanded in the AML microenvironment, exhibit a non-activated phenotype, producing low levels of IFN-α in response to TLR stimulation. The persistence of TAPDCs supports Treg expansion and the maintenance of an immunosuppressive niche (*10*, *47*).

In pDC-AML, this immunosuppressive role becomes even more evident. TAPDCs release leukemic antigens while failing to mount an effective type I IFN response, thereby facilitating immune escape by leukemic clones. These pDCs also skew T cell differentiation toward Treg and Th2 profiles, a shift supported by their expression of IDO and PD-L1. Additionally, elevated levels of transforming growth factor (TGF) β and TNF in the leukemic microenvironment can inhibit *IRF-7*, a key transcription factor required for IFN-α production, thereby reinforcing the dysfunctional state of TAPDCs (*10*, *44*, *45*).

Furthermore, studies show that reduced levels of cDCs at diagnosis and a predominance of pDCs during remission are frequently observed in AML (*48*). This imbalance suggests a disrupted DC compartment, where a higher ratio of pDCs to moDCs may correlate with immunosuppression and poor clinical outcomes (*28*). According to Xiao *et al.*, the loss of pDC differentiation is predictive of residual disease post-induction therapy, positioning pDC profiling as a potential tool for risk stratification. Notably, in AML-M4/M5, pDC infiltration has been linked to disease progression. Under the leukemic microenvironment, pDCs may differentiate into tumor-associated dendritic cells, contributing to immune evasion and treatment resistance (*27*).

## DC-based therapies

Over the past decades, DC-based vaccines have emerged as a promising immunotherapeutic strategy in the treatment of hematologic malignancies, including AML. Continuous research has explored advanced methodologies to enhance their clinical potential and overcome immune resistance mechanisms (*49*). While allogeneic hematopoietic stem cell transplantation remains a valuable treatment option, its use is often limited by severe complications such as GVHD, contributing to significant morbidity and mortality (*50*, *51*). Table 1 summarizes the main DC-based vaccination strategies currently under clinical investigation.

**Table 1:** Relevant clinical trials available on vaccination therapy in acute myeloid leukemia

**Vaccine type**	**Vaccine antigen**	**Doses**	**Phases endpoint**	**Results**	**Authors**
DC	WT1	4 (2 every 2 months)	Antileukemic response	Relapse reduction rate of 25%	Anguille S *et al.* (*57*)
Allogeneic DC	WT1, PRAME	4	Phase I	Tolerated treatment Increased overall survival Development of cellular and humoral immune response	Van de Loosdrecht A *et al.* (*58*)
Genetically modified DC (gmDC)	MUC1, flagellin, SOCS1	1	Phase I – II gmDCs elicited potent TAA-specific CTL responses *in vitro*	Phase I: safe in acute leukemia patients and yielded improved survival rate. Phase II: complete remission rate of 83% in 12 relapsed AML patients.	Wang D *et al.* (*52*)
DC	WT1, PRAME	4, every 6 weeks	Improved immune response rate	Stable disease	Eckl J *et al.* (*59*)
TLR7/8-matured DCs transfected with RNA	WT1, PRAME, CMVpp65	10	Phase I Immune response	TLR7/8-matured DCs to AML patients in CR at high risk of relapse was feasible and safe and resulted in induction of antigen-specific immune responses.	Lichtenegger FS *et al.* (*24*)
moDC autologous	-	5 (days 1, 7, 14, 21, 35)	Phase I/II	No severe adverse event was documented. prolonged overall survival	Chevallier P *et al.* (*60*)
Autologous RNA-loaded mature dendritic cell (mDC)	WT, PRAME	1 (4 week, 6 week). 1 monthly dose for 2 years	-	Treatment was well tolerated; Eleven of 20 patients (55%) remained in CR; Overall survival at five years was 75% (95% CI: 50–89), with 70% of patients ≥60 years of age being long-term survivors	Floisand Y *et al.* (*61*)
Allogeneic Leukemia-Derived Dendritic Cell	-	4 fortnightly; Reinforcement (week 14 and 18).	Improved immune response rate	Increase in the number of circulating antigen-presenting DCs Vaccination improve and induce maturation of DC (cDC1, cDC2 and pDC), presentation to tumor-reactive T cells	Van Zeeburg H *et al.* (*62*)
DC/AML fusion vaccine		Mean fusion vaccine dose was 3,7 x10^6^ cell 2 doses with a 3-week interval.	Phase I Result	20 effectively received the vaccine; adverse reactions included mild local reactions; 5 cases of GVHD possibly related to vaccination; 45% had high-risk disease, and 14 remained in complete remission, with late relapses (3–5 years); Median overall survival was 50 months after transplantation. The vaccine has proven to be safe, feasible, and potentially effective	Liegel J *et al.* (*63*)
DC - dendritic cell. AML - acute myeloid leukemia. TAA - tumor-associated antigen. CTL - cytotoxic T cell. CR - complete remission. GVHD - graft-*versus*-host disease.					

Dendritic cell vaccines can be generated from various cellular sources, commonly leukemia-derived DCs (AML-DCs) and monocyte-derived DCs (moDCs). However, a central challenge in vaccine development lies in selecting appropriate antigenic targets, due to the inherent heterogeneity of leukemic clones. To navigate this complexity, leukemic antigens are generally classified into two main categories: leukemia-associated antigens (LAAs), which are overexpressed or aberrantly expressed in leukemic cells (*e.g*., Wilms’ tumor protein 1 – WT1), and leukemia-specific antigens (LSAs), which result from somatic mutations and include true neoantigens, such as Nucleophosmin 1 (NPM1) (*44*, *52*).

Despite encouraging results from preclinical and early-phase clinical trials, several barriers still hinder the complete optimization and clinical efficacy of DC vaccines in AML. These include the difficulty in identifying universal, immunogenic antigens due to disease heterogeneity, challenges in standardizing DC generation protocols, and determining the most effective routes of administration. Furthermore, the immunosuppressive nature of the AML microenvironment, characterized by Treg expansion, dysfunctional antigen presentation, and inhibitory cytokine signaling, remains a significant obstacle that may substantially diminish the vaccine’s therapeutic potential (*1*, *50*).

The primary antigens utilized in DC-based vaccines are WT1 and PRAME, often combined with additional targets to enhance applicability across patients; however, these approaches still fail to fully address tumor heterogeneity. Several vaccine platforms are under development, including autologous, allogeneic, and genetically modified DCs, each exhibiting distinct immunogenic profiles and clinical potential. These strategies have demonstrated increased interferon production and expansion of cytotoxic CD8+ T lymphocytes, indicating effective activation of antitumor immune responses. Despite encouraging results, the lack of methodological standardization, limited sample sizes, and scarcity of advanced-phase randomized trials continue to hinder the establishment of definitive evidence of clinical efficacy. Nevertheless, DC vaccines remain a promising and safer alternative to CAR-T cell or bispecific T-cell engager antibody therapies, which, although highly effective, are frequently associated with severe toxicities such as cytokine release syndrome, neurotoxicity, and tumor lysis syndrome (*53*). Moreover, many CAR-T targets, including CD33, CD117, and CD123, are also expressed on hematopoietic stem cells, raising the risk of severe and potentially fatal myeloablation (*54*).

Among the barriers to implementing DC vaccines, one difficulty is the complexity and high cost of individualized production, which requires leukapheresis, cell culture, genetic manipulation, or fusion with leukemic blasts, in addition to rigorous quality controls, making the process time-consuming and dependent on specialized infrastructure. The time required for vaccine preparation may not coincide with the ideal therapeutic window, especially in post-transplant patients, who may experience toxicities or relapse before vaccination (*55*). Another relevant challenge is the risk of interference from immunosuppression and the potential for induction of GVHD, which requires a careful balance between immunological efficacy and safety. Tumor heterogeneity and antigen loss also limit the durability of the immune response, while the absence of predictive biomarkers and large-scale clinical studies prevents adequate patient selection and regulatory approval (*56*). Finally, the limited scalability and difficulty of integration into hospital routines reinforce the need to develop more standardized, accessible, and “off-the-shelf” platforms to enable large-scale clinical use.

## Conclusion

In summary, DCs, particularly pDCs, play a central role in modulating the immune response in hematological malignancies, such as acute myeloid leukemia. Their ability to present leukemic antigens and coordinate the activation of T lymphocytes positions them as critical mediators between immunity and tumor tolerance. However, mechanisms such as the downregulation of MHC molecules, the expansion of regulatory T cells, T lymphocyte exhaustion, and immunosuppressive remodeling of the microenvironment compromise their function, favoring immune evasion and disease progression. Thus, understanding the interactions between DCs and the leukemic microenvironment is crucial for elucidating their role in both resistance and therapeutic response.

In this context, DC-based immunotherapies, especially dendritic cell vaccines, emerge as a promising alternative to conventional immunological approaches, such as CAR-T therapies, offering the potential for personalization and lower toxicity. Although challenges persist, including tumor heterogeneity, the ideal selection of antigens, and the influence of the immunosuppressive microenvironment, advances in the phenotypic and functional characterization of DCs pave the way for more effective strategies to reactivate antitumor immunity. A deeper understanding of their biology and regulatory mechanisms will be crucial to consolidating the role of DC vaccines as a viable therapeutic tool, capable of improving prognosis and clinical outcomes in AML and related hematological malignancies.

## Data Availability

No data was generated during this study, so data sharing statement is not applicable to this article.
